# Oxidative Stress and Antioxidant Status in High-Risk Prostate Cancer Subjects

**DOI:** 10.3390/diagnostics10030126

**Published:** 2020-02-27

**Authors:** Sanjeev Shukla, Janmejai K. Srivastava, Eswar Shankar, Rajnee Kanwal, Akbar Nawab, Haripaul Sharma, Natarajan Bhaskaran, Lee E. Ponsky, Pingfu Fu, Gregory T. MacLennan, Sanjay Gupta

**Affiliations:** 1 Department of Urology, Case Western Reserve University, School of Medicine, Cleveland, OH 44106, USA; sanjeev.shukla@case.edu (S.S.); jksrivastava@lko.amity.edu (J.K.S.); eswar.shankar@case.edu (E.S.); rajnee.fnu@case.edu (R.K.); akbarnawab@gmail.com (A.N.); haripaul.sharma@gmail.com (H.S.); natarajan.bhaskaran@case.edu (N.B.); Lee.Ponsky@uhhospitals.org (L.E.P.); 2 The Urology Institute, University Hospitals Cleveland Medical Center, Cleveland, OH 44106, USA; gtm2@case.edu; 3 Present Address: Department of Anatomy and Cell Biology, University of Florida, Gainesville, FL 32611 USA; 4 Department of Population and Quantitative Health Sciences, Case Western Reserve University, Cleveland, OH 44106, USA; pxf16@case.edu; 5 Department of Pathology, Case Western Reserve University, Cleveland, OH 44106, USA; 6 Department of Nutrition, Case Western Reserve University, Cleveland, OH 44106, USA; 7 Division of General Medical Sciences, Case Comprehensive Cancer Center, Cleveland, OH 44106, USA; 8 Department of Urology, Louis Stokes Cleveland Veterans Affairs Medical Center, Cleveland, OH 44106, USA

**Keywords:** oxidative stress 1, inflammation 2, antioxidant enzymes 3, DNA damage 4, prostate cancer 5

## Abstract

The oxidant/antioxidant balance has been implicated in the pathophysiology of prostate cancer. We investigated oxidative damage and antioxidant status in high-risk prostate cancer subjects. Reduced glutathione (GSH) levels were measured in erythrocytes, 8-hydroxydeoxyguanosine (8-OHdG) in leukocytes and plasma levels of catalase (CAT), glutathione peroxidase (GSH-Px), glutathione reductase (GSH-R), glutathione S-transferase (GST), superoxide dismutase (SOD), and lipid peroxide products were measured in high-risk and age-matched healthy subjects. Serum PSA levels were significantly higher (*p* < 0.0001) in high-risk subjects, whereas GST (*p* < 0.0001) and GSH (*p* < 0.002) were higher in healthy controls. Levels of 8-OHdG, an oxidized nucleoside of DNA, were significantly increased (*p* < 0.0001) in high-risk subjects. No marked difference in the levels of CAT (*p* = 0.237), GSH-Px (*p* = 0.74), GSH-R (*p* = 0.344), SOD (*p* = 0.109), and lipid peroxide products (*p* = 0129) were observed between two groups. Pearson’s correlation between GST and PSA (r = −0.69 (*p* < 0.0001)), GST and 8-OHdG (r = −0.62 (*p* < 0.0004)), GSH and 8-OHdG (r= −0.39 (*p* = 0.038)), and CAT and GSH-Px (r= −0.33 (*p* = 0.04)) were found to be negatively correlated, whereas 8-OHdG and PSA were positively associated (r= 0.57 (*p* < 0.002). These results indicate a significant role of oxidative damage in prostate carcinogenesis, particularly during the early stages of development. In conclusion, our data support the importance of antioxidant defense as a valuable diagnostic and/or prognostic marker in prostate cancer.

## 1. Introduction

Prostate cancer is an important public health problem, particularly in Western countries with trends towards increase in aging population [1,2]. The etiology and the risk factors of prostate malignancy is not well understood, however, certain risk factors are frequently associated to its development. Non-modifiable risk factors include age, race/ethnicity, genetic factors, and family history [3,4], whereas environmental factors, diet, and lifestyle are some modifiable risk factors for prostate cancer [5,6].

Reports suggest that prostate cancer is frequently related with a shift in the oxidant/antioxidant balance resulting in increased oxidative stress [7,8]. Accumulating evidence suggest that intracellular production of deleterious molecules of oxidative damage plays a critical role in aging and age-related diseases such as prostate cancer [9,10]. Reactive oxygen species (ROS), such as hydroxyl radicals, superoxide anion and hydrogen peroxides are capable of inducing lipid peroxidation and genomic DNA damage altering the activity of sulfhydryl (SH)-dependent enzymes [11]. Reports suggest age-related molecular changes in the prostate as a result of oxidative DNA damage induced by hydroxyl radicals. Progressive age-related DNA damage and higher accumulation of 8-oxo-2′-deoxyguanosine (8-OHdG) has been markedly increased in clinical specimens of prostate cancer, compared to benign tissue [12,13]. We have previously demonstrated that chronic inflammation causes premalignant and malignant changes in the prostate as a result of increased oxidative stress, ROS generation, and DNA damage along with alteration in antioxidant enzyme activity [14,15].

The cellular antioxidant system that controls ROS production includes enzymes such as glutathione peroxidase (GSH-Px), superoxide dismutase (SOD), and catalase (CAT), which reduce hydroperoxides while oxidizing cellular glutathione [16,17]. The oxidized glutathione (GSSG) is recycled to GSH by glutathione reductase (GSH-R) utilizing NADPH to reduce GSSG. NADPH is regenerated by glucose-6-phosphate dehydrogenase (G6PDH) in the hexose monophosphate shunt. In addition, glutathione S-transferase (GST), a set of isozymes, catalyzes intracellular detoxification reactions by conjugating glutathione with ROS, resulting in the generation of less toxic products [18,19]. We have shown that the GST isozyme, GST-pi, protects against oxidative DNA damage to prostate epithelial cells. Loss of GST-pi is an early event demonstrated in prostate cancer [19,20].

The relationship of oxidative stress to the development of cancer has been a subject of frequent discussion, with a few studies reporting altered pro-oxidant–antioxidant status in clinical prostate cancer specimens, rodent models, and prostate cell lines [9,10,11]. However, the data regarding the antioxidant status, lipid peroxidation, and DNA damage and their correlation in plasma/serum specimens in subjects with high-risk for prostate cancer has not been elucidated. In this study, we investigate whether antioxidant enzyme activity, lipid peroxidation, and oxidative DNA damage may be developed as a biomarker to identify men who are at a higher risk of developing prostate cancer.

## 2. Materials and Methods

### 2.1. Study Subjects

A total of 40 men were included in the study in the age range of 52–84 years without any prior drug or treatment involvement. Patients were recruited from the Urology clinic of the University Hospitals Cleveland Medical Center between January 2008 and May 2011. Twenty subjects were selected who were diagnosed having precursor high-grade intraepithelial neoplasia (HGPIN) lesions confirmed by needle biopsy and serum PSA > 4.0 ng/mL and abnormality observed in the prostate during digital rectal exam or transrectal ultrasonography. 20 age-matched men within the same age group designated as controls were recruited in the study having serum PSA < 4 ng/mL, normal digital rectal exam without urinary symptoms and diagnosis of benign prostatic hyperplasia (BPH) or prostatitis. Detailed information such as demographic, disease history, and family history of cancer was documented. Specific exclusion criteria considered for the present study were those having a history of cerebrovascular or ischemic heart diseases, chronic obstructive pulmonary disease, psoriasis, pelvic inflammatory disease, multiple sclerosis, severe arthritis, lupus, Hashimoto thyroiditis, inflammatory bowel disease, renal insufficiency, and diabetes mellitus. The participants were not allowed to take non-steroidal anti-inflammatory medications, antioxidant, or vitamin supplements and consume alcohol, at the time of the study. Written informed consent was acquired from all participants in the study before the collection of blood specimens. The study was approved on August 28, 2008 by the Institutional Review Board of Case Comprehensive Cancer Center (CASE11807) and clinicaltrials.gov identifier NCT00898274. 

### 2.2. Sample Preparation.

Patients were first required to fast overnight, followed by blood samples drawn from the antecubital vein in EDTA glass tubes. These samples were centrifuged for 10 min at 4000× *g* at 4 °C. Plasma and buffy coats were removed. The remaining erythrocyte pellet was washed with isotonic saline and lysed with cold distilled water (1:4). Following lysis, the samples were stored in a 4 °C refrigerator for 15 min. Cell debris was removed by centrifugation (2000× *g* for 10 min). Plasma samples and erythrocyte fraction were stored at −80 °C until assayed. We performed all antioxidant and oxidants profiling using assay kits and the patients’ samples were repeated twice in triplicates. All biochemical assays were performed as mentioned in manufacturer’s protocol.

### 2.3. Lipid Hydroperoxide Assay

Lipid hydroperoxides were measured spectrophotometrically in the plasma using Lipid Hydroperoxide (LPO) Assay Kit (Cat# 705002) from Cayman Chemical Company (Ann Arbor, MI, USA), which directly utilizes the redox reactions with ferrous ions. Chloroform extraction was performed to extract lipid hydroperoxides and this extract was directly used to determine LPO levels.

### 2.4. DNA Damage Assay

To quantify 8-OHdG levels using the ELISA assay, DNA isolated from the buffy coat was first processed to single stranded DNA using OxiSelect™ Oxidative DNA damage ELISA kit, Cell Biolabs, Inc. (San Diego, CA, USA) as per vendor’s instructions. The 8-OHdG standard curve was then used to determine 8-OHdG levels in the specimen by comparing its absorbance.

### 2.5. Glutathione Pathway Assays

Assay kits for determination of glutathione concentration (Cat# 703302) from erythrocytes, and the plasma activities of glutathione peroxidase (GSH-Px) (Cat# 703102), glutathione S-transferase (GST) (Cat# 703302) and glutathione reductase (GR) (Cat# 703202) purchased from Cayman Chemical (Ann Arbor, MI) were performed according to manufacturer’s protocol using appropriate standards.

### 2.6. Catalase Activity Assay

Catalase enzyme is involved in the detoxification of hydrogen peroxide and was measured in the plasma from control and high-risk subjects using Catalase assay kit (Cat# 707002) from Cayman Chemical, Ann Arbor, MI, USA. The formaldehyde produced during catalytic reaction was spectrophotometrically measured with 4-amino-3-hydrazino-5-mercapto-1, 2, 4-triazole (Purpald) as the chromogen.

### 2.7. Superoxide Dismutase Activity Assay

Superoxide dismutase activity was measured in the plasma from control and high-risk subjects using Superoxide Dismutase assay kit (Cat# 706002) from Cayman Chemical (Ann Arbor, MI, USA), which quantifies the activities of all three types of Cu/Zn-, Mn-, and Fe-SOD measuring the dismutation of superoxide radicals generated by xanthine oxidase and hypoxanthine.

### 2.8. Prostate Specific Antigen Assay

PSA was assayed from the serum using PSA ELISA kit (Cat# 07BC-1019) from MP Biomedicals (Irvine, CA, USA) following vendor’s instruction.

### 2.9. Statistical Analysis

Data was summarized as the mean, standard deviation (std. dev.), as well as a box plot. The difference of antioxidant enzyme activities and DNA/lipid damage between healthy controls and high-risk patients was examined using a *t*-test. The association between any two continuous parameters was estimated using Pearson correlation coefficient and illustrated using a scatter plot. All tests were two-tailed and a *p*-value less than 0.05 was considered to be statistically significant.

## 3. Results

The serum PSA profile of control and high-risk subjects are summarized in Supplemental Table S1. Serum PSA levels in control subjects (*n* = 20) ranged from 0.84 to 6.34 ng/mL; average 2.598 ± 1.53 ng/mL. In high-risk group (*n* = 20) serum PSA ranged from 7.32 to 34.44 ng/mL; average 17.315 ± 7.03 ng/mL. Serum PSA levels were significantly higher (*p* < 0.0001) in high-risk subjects, compared to the control group.

**Figure 1:**
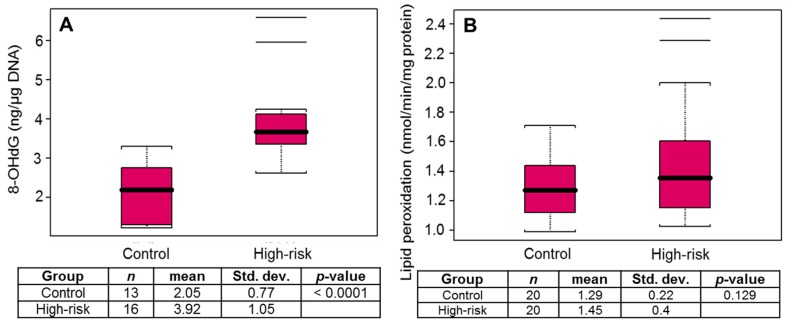
Box plot for (**A**) 8-OHdG and (**B**) lipid peroxidation in healthy controls and high-risk subjects for prostate cancer. Black bar = median, red box = 25th to 75th percentiles, Bars = entire range. The horizontal lines beyond the bars are outliers or whiskers are drawn individually.

In next set of experiments, 8-hydroxydeoxyguanosine (8-OHdG) levels in the DNA isolated from the buffy coat and plasma levels of lipid peroxide products from control and high-risk subjects were measured. Data obtained after analysis was represented as the mean, standard error, and a box plot. Levels of 8-OHdG, an oxidized nucleoside of DNA, were significantly increased (*p* < 0.0001) in high-risk subjects versus the control group (Figure 1A). A modest increase with no significant difference in the levels of lipid peroxide products was observed between the groups (Figure 1B).

**Figure 2:**
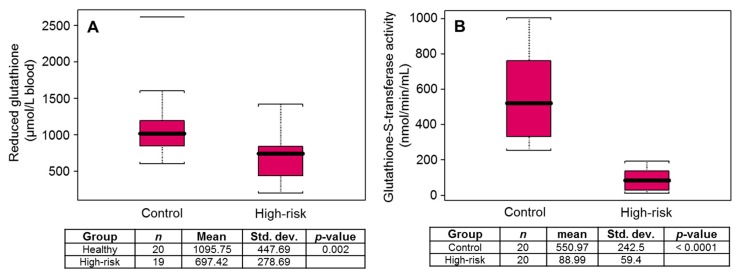
Box plot for (**A**) reduced glutathione levels, and (**B**) glutathione S-transferase activity in healthy controls and high-risk subjects for prostate cancer. Black bar = median, red box = 25th to 75th percentiles, Bars = entire range. The horizontal lines beyond the bars are outliers or whiskers are drawn individually.

Next reduced glutathione (GSH) levels in erythrocytes and plasma GST activity were measured in the samples obtained from control and high-risk subjects. GSH levels were higher (*p* < 0.002) in the control subjects, compared to the high-risk group (Figure 2A). Similarly, plasma GST levels were significantly higher (*p* < 0.0001) in the control group versus high-risk subjects (Figure 2B).

**Figure 3:**
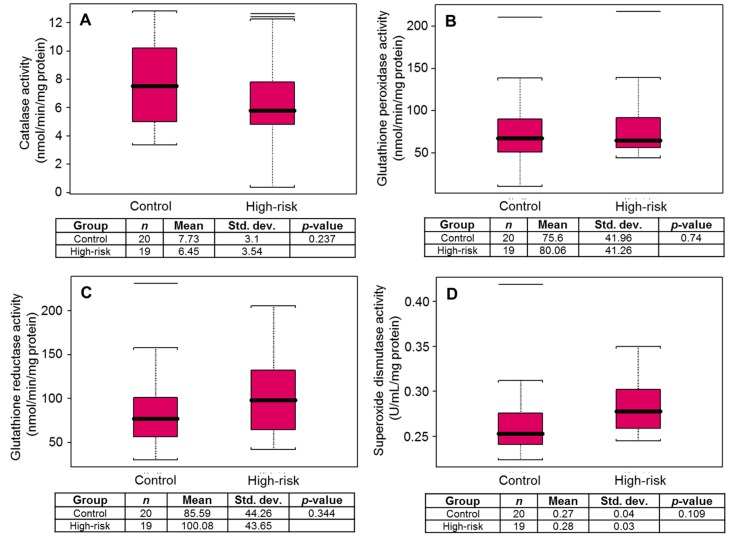
Box plot for (**A**) catalase, (**B**) glutathione peroxidase, (**C**) glutathione reductase, and (**D**) superoxide dismutase activity in healthy controls and high-risk subjects for prostate cancer. Black bar = median, red box = 25th to 75th percentiles, Bars = entire range. The horizontal lines beyond the bars are outliers or whiskers are drawn individually.

Thereafter the plasma levels of catalase (CAT), glutathione peroxidase (GSH-Px), glutathione reductase (GSH-R), and superoxide dismutase (SOD) were analyzed. No significant difference in the levels of CAT (*p* = 0.237), GSH-Px (*p* = 0.74), GSH-R (*p* = 0.344), and SOD (*p* = 0.109) activity were noted in high-risk subjects, compared to healthy controls (Figure 3A–D).

**Figure 4:**
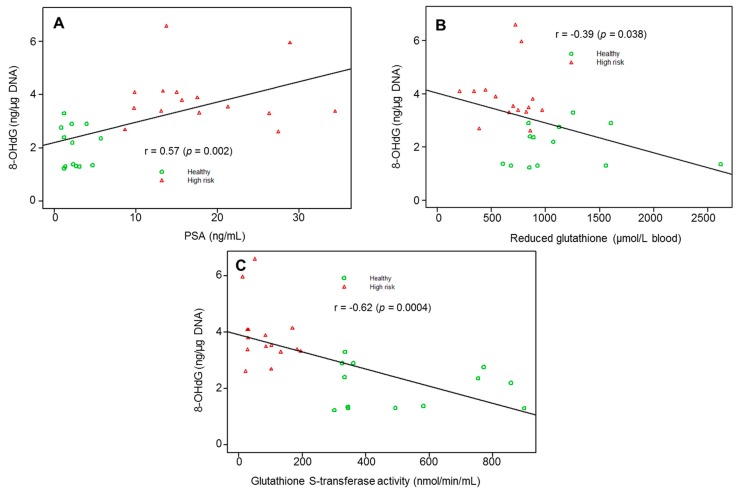
Correlation between (**A**) 8-OHdG versus serum PSA, (**B**) 8-OHdG versus erythrocyte reduced glutathione (GSH), and (**C**) 8-OHdG versus glutathione S-transferase activity in healthy controls and high-risk subjects for prostate cancer. The association was estimated using the Pearson correlation coefficient (r) and illustrated using a scatter plot. *p*-value < 0.05 is considered significant.

Next an association between oxidative DNA damage and antioxidant enzymes; 8-OHdG and PSA levels were determined using the Pearson correlation coefficient. A significant positive correlation between 8-OHdG and PSA (r = 0.57 (*p* = 0.002)) was observed; whereas negative correlation between the association of 8-OHdG and GSH (r = −0.39 (*p* = 0.038)), and 8-OHdG and GST (r= −0.62 (*p* = 0.0004)) was noted between the groups (Figure 4A–C).

**Figure 5:**
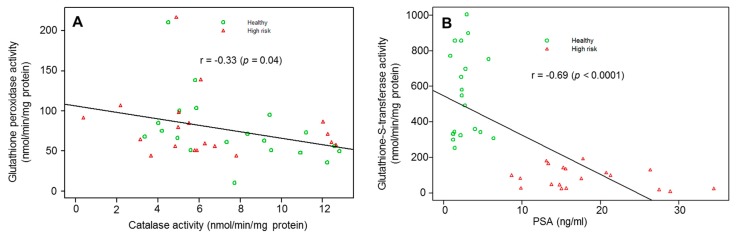
Correlation between (**A**) glutathione peroxidase activity versus catalase activity, (**B**) glutathione S-transferase activity versus PSA in healthy controls and high-risk subjects for prostate cancer. The association was estimated using the Pearson correlation coefficient (r) and illustrated using a scatter plot. *p*-value < 0.05 is considered significant.

In addition, a negative association between GSH-Px and CAT activity (r= −0.33 (*p* = 0.04)) and GST and PSA (r = −0.69 (*p* < 0.0001)) was noted between the groups (Figure 5A,B).

## 4. Discussion

Oxidative stress and accumulated DNA damage increases the risk of prostate cancer [21]. The present data outline that chronic inflammation mediated ROS production might play an important role in causing DNA damage leading to neoplastic transformation in prostate epithelial cells. Our previous prospective 5-year study looking at needle biopsy specimens established a correlation between intraprostatic inflammation leading to neoplastic changes in prostatic tissue [14,15]. The results in this study demonstrated a marked increase in DNA damage product 8-OHdG (*p* < 0.0001), insignificantly increased levels of oxidized lipid products (*p* = 0.129), SOD (*p* = 0.109), GSH-Rx (*p* = 0.74), and GSH-Px (*p* = 0.74); significant downregulation in reduced glutathione (GSH; *p* < 0.002) and glutathione s-transferase (*p* < 0.0001); whereas an insignificant decrease in CAT activity (*p* = 0.237) in the plasma of high-risk subjects occurred compared to healthy controls.

8-OHdG is the most commonly used biomarker to measure oxidative DNA damage [12,22]. Studies have shown that 42% of men aged 55–80 years exhibit prostate DNA damage assessed by 8-OHdG levels [13,23]. Whereas previous published literature on 8-OHdG have examined tissue and urinary levels of this adduct [13,23], studies using leukocytes from blood samples have been limited. To our knowledge, this is a unique study assessing the levels of oxidative DNA damage, 8-OHdG from the blood of healthy and high-risk subjects for prostate cancer. A significant association between PSA levels and 8-OHdG was noted in the high-risk group. We could suggest that plasma 8-OHdG levels might be a significant biomarker of oxidative damage in high-risk subjects and prostate cancer patients.

Among non-protein thiols, GSH plays a key role in maintaining the intracellular antioxidant defense by scavenging ROS and regenerating other antioxidant molecules [24]. In the cytoplasm, micromolar concentrations of reduced GSH efficiently dissociate hydrogen peroxide. This process results in the increase in the level of oxidized GSH (GSSG), which is converted back to GSH by GSH-R in an NADPH-dependent redox cycle, to maintain sufficient intracellular GSH levels. Our study demonstrated a significant decrease in the levels of reduced GSH in high-risk subjects compared to healthy controls. These findings are in disagreement with another study that showed increased antioxidant defense during prostate cancer progression and increase in GSH in cancer cells [17]. There was also a significant negative association noted between GSH and 8-OHdG (r = −0.39). The possible explanation for this is that cellular ROS generation might serve as a switch where non-protein thiols are downregulated in neoplastic transformation and increases as a compensatory mechanism in cancer.

Lipid peroxidation is a free radical reaction that involves oxidative conversion of polyunsaturated fatty acids to malondialdehyde (MDA) or other lipid hydroperoxide products such as 4-hydroxy-2-nonenal (4HNE), 4-oxo-2-nonenal and acrolein [25,26]. MDA remains the most mutagenic molecule, whereas 4HNE appears to be the most toxic byproduct of lipid peroxidation. These peroxidation products conjugate with intracellular GSH and GST facilitating their detoxification [27]. In the present study, lipid hydroperoxide content was increased although insignificantly in high-risk subjects, compared to healthy controls. These results, in part, are in accordance with previous studies confirming an increase in lipid peroxidation in prostate cancer compared to benign tissue [28,29]. This is plausible as alteration in lipid peroxidation might trigger changes in cellular antioxidant defense system, in particular glutathione metabolizing enzymes during cancer progression. Our results demonstrated a modest increase in lipid peroxide products, which may be due to either lower production of lipid peroxidation molecules in high-risk subjects or sensitivity of the assay, however their levels have been demonstrated to significantly increase in prostate cancer patients [7,10].

Direct elimination of ROS is mediated by SOD, GSH-Px, and CAT, which are considered as primary antioxidant caretaker enzymes [16,17]. These enzymes protect cells against deleterious effect of ROS produced during normal metabolism and during oxidative stress. SOD catalyzes the dismutation of superoxide radical to form H_2_O_2_. The H_2_O_2_ generated is disintegrated to water molecules by CAT or GSH-Px. During GSH-Px catalyzed decomposition of H_2_O_2_, the GSH is converted to GSSG, which is then recycled to GSH by GSH-R. In addition, GST utilizes GSH as a cofactor for the detoxification of organic hydroperoxides and other electrophiles derived as by-products from lipid peroxidation. Our results revealed that CAT activity was modestly decreased in high-risk subjects, compared to healthy controls. These results were in agreement with other findings showing lower CAT and GSH-Px activity in prostate cancer, compared to benign tissue. A significant negative association was noted between CAT and GSH-Px activity in our present study. Bostwick et al. (2000) reported a decrease in CAT expression in prostate cancer implicating oxidative DNA damage [9]. We observed modestly higher SOD activity in high-risk subjects. Studies suggest higher SOD levels without compensatory increase in CAT has deleterious cellular effects [30]. This imbalance could be due to persistent low-grade inflammation, which might lead to a subsequent increase in SOD levels. A study by Blum and Fridovich (1985) demonstrates that loss of GSH-Px activity is observed in oxidative stress conditions induced by superoxide anions, and toxic molecules are generated during lipid peroxidation [31]. This implicates a high production of superoxide radical generated during cancer can inactivate CAT and GSH-Px and may be a probable reason for the decrease activity of these enzymes in prostate carcinogenesis.

The non-protein thiol GSH, and GSH-R maintains an optimum cellular redox potential through the inactivation of H_2_O_2_. Studies have demonstrated the contribution of GSH and GSH-R in cellular protection against ROS in prostate cancer cells [32,33]. Kumar et al. (2008) have reported an increase in GSH-R and inhibition of GSH activities in the human prostate cancer cell lines PC3, LNCaP, and DU145, when compared with primary cell cultures of benign and malignant human prostatic tissue [34]. In the present study, a marked decrease in GSH and insignificant increase in GSH-R was noted in high-risk subjects, compared to healthy controls. Thus, GSH and GSH-R might contribute to protection against ROS.

Reports suggest that GSTs possess a caretaker function, protecting cells against damage induced by electrophiles and free radicals [35]. Loss of GST has been shown to increase the inflammatory response in various tissues as a result of infiltration of immune cells [36,37]. Others and we have previously demonstrated that GST elicits protection against oxidative DNA damage and stress in human prostate epithelial cells [18,20]. The present data confirm the previous results in demonstrating a significant decrease in plasma GST activity in high-risk subjects, compared to healthy individuals. Loss of GST, especially the GST-pi class has been shown in prostate epithelial cells during the early stages of neoplastic development and cancer [19,38]. Considering GST-pi as an antioxidant enzyme, its loss initiated by epigenetic/genetic alterations could lead to tissue damage and promote carcinogenesis [18,19,35,36,37]. This hypothesis is strongly supported by our present findings. Firstly, significant low GST activity observed in the plasma of high-risk subjects, which can be attributed to the depletion of the antioxidant defense system and overwhelming production of free radicals. Secondly, a strong negative association between GST and PSA (r = −0.69 (*p* < 0.0001)) and higher levels of 8-OHdG and lipid peroxidation products in the blood of high-risk subjects facilitating disease progression. The circulating antioxidant defense enzymes might be depleted in an attempt to counterbalance oxidative stress that occurs during aging and might also play a role in prostate carcinogenesis.

In this study, we utilized standard ELISA assays to measure changes in various antioxidant enzyme molecules and oxidative DNA damage products in the blood samples. Measurement of antioxidant protein by their activity provides more specific diagnostic information in the plasma, with consistent and reproducible results, that can be duplicated in other laboratories. While this study has the limitation of the restricted sample size, additional studies with large sample sizes are necessary to confirm the precise association between oxidative stress and prostate cancer. Further studies are required to determine whether diagnostic biomarkers of prostate cancer can be developed using oxidative stress-related parameters.

## 5. Conclusions

In conclusion, the present study demonstrated that increased levels of oxidative damage and changes in the antioxidant defense system in high-risk subjects might have a possible link between oxidative stress and prostate cancer. The results of this study could be useful in risk stratification and in devising nomograms for early prevention and treatment of prostate cancer.

## Supplementary Materials

The following are available online at https://www.mdpi.com/2075-4418/10/3/126/s1.

## References

[1] Bray F., Ferlay J., Soerjomataram I., Siegel R.L., Torre L.A., Jemal A. (2018). Global cancer statistics 2018: GLOBOCAN estimates of incidence and mortality worldwide for 36 cancers in 185 countries. Ca A Cancer J. Clin..

[2] DeSantis C.E., Miller K.D., Dale W., Mohile S.G., Cohen H.J., Leach C.R., Goding Sauer A., Jemal A., Siegel R.L. (2019). Cancer statistics for adults aged 85 years and older, 2019. Ca A Cancer J. Clin..

[3] Zeng C., Wen W., Morgans A.K., Pao W., Shu X.O., Zheng W. (2015). Disparities by Race, Age, and Sex in the Improvement of Survival for Major Cancers: Results From the National Cancer Institute Surveillance, Epidemiology, and End Results (SEER) Program in the United States, 1990 to 2010. JAMA Oncol..

[4] Krishna S., Fan Y., Jarosek S., Adejoro O., Chamie K., Konety B. (2017). Racial Disparities in Active Surveillance for Prostate Cancer. J. Urol..

[5] Peisch S.F., Van Blarigan E.L., Chan J.M., Stampfer M.J., Kenfield S.A. (2017). Prostate cancer progression and mortality: A review of diet and lifestyle factors. World J. Urol..

[6] Adjakly M., Ngollo M., Dagdemir A., Judes G., Pajon A., Karsli-Ceppioglu S., Penault-Llorca F., Boiteux J.P., Bignon Y.J., Guy L. (2015). Prostate cancer: The main risk and protective factors-Epigenetic modifications. Ann. D’endocrinol..

[7] Battisti V., Maders L.D., Bagatini M.D., Reetz L.G., Chiesa J., Battisti I.E., Goncalves J.F., Duarte M.M., Schetinger M.R., Morsch V.M. (2011). Oxidative stress and antioxidant status in prostate cancer patients: Relation to Gleason score, treatment and bone metastasis. Biomed. Pharmacother..

[8] Arsova-Sarafinovska Z., Eken A., Matevska N., Erdem O., Sayal A., Savaser A., Banev S., Petrovski D., Dzikova S., Georgiev V. (2009). Increased oxidative/nitrosative stress and decreased antioxidant enzyme activities in prostate cancer. Clin. Biochem..

[9] Bostwick D.G., Alexander E.E., Singh R., Shan A., Qian J., Santella R.M., Oberley L.W., Yan T., Zhong W., Jiang X. (2000). Antioxidant enzyme expression and reactive oxygen species damage in prostatic intraepithelial neoplasia and cancer. Cancer.

[10] Aydin A., Arsova-Sarafinovska Z., Sayal A., Eken A., Erdem O., Erten K., Ozgok Y., Dimovski A. (2006). Oxidative stress and antioxidant status in non-metastatic prostate cancer and benign prostatic hyperplasia. Clin. Biochem..

[11] Oberley T.D., Zhong W., Szweda L.I., Oberley L.W. (2000). Localization of antioxidant enzymes and oxidative damage products in normal and malignant prostate epithelium. Prostate.

[12] Ohtake S., Kawahara T., Ishiguro Y., Takeshima T., Kuroda S., Izumi K., Miyamoto H., Uemura H. (2018). Oxidative stress marker 8-hydroxyguanosine is more highly expressed in prostate cancer than in benign prostatic hyperplasia. Mol. Clin. Oncol..

[13] Malins D.C., Johnson P.M., Wheeler T.M., Barker E.A., Polissar N.L., Vinson M.A. (2001). Age-related radical-induced DNA damage is linked to prostate cancer. Cancer Res..

[14] MacLennan G.T., Eisenberg R., Fleshman R.L., Taylor J.M., Fu P., Resnick M.I., Gupta S. (2006). The influence of chronic inflammation in prostatic carcinogenesis: A 5-year followup study. J. Urol..

[15] Chen W., Jia L., Gupta S., MacLennan G. (2019). The Role of Chronic Inflammation in Prostate Carcinogenesis: A Follow-Up Study. Ann. Urol. Oncol..

[16] He L., He T., Farrar S., Ji L., Liu T., Ma X. (2017). Antioxidants Maintain Cellular Redox Homeostasis by Elimination of Reactive Oxygen Species. Cell. Physiol. Biochem. Int. J. Exp. Cell. Physiol. Biochem. Pharmacol..

[17] Chaiswing L., Zhong W., Oberley T.D. (2014). Increasing discordant antioxidant protein levels and enzymatic activities contribute to increasing redox imbalance observed during human prostate cancer progression. Free Radic. Biol. Med..

[18] Mian O.Y., Khattab M.H., Hedayati M., Coulter J., Abubaker-Sharif B., Schwaninger J.M., Veeraswamy R.K., Brooks J.D., Hopkins L., Shinohara D.B. (2016). GSTP1 Loss results in accumulation of oxidative DNA base damage and promotes prostate cancer cell survival following exposure to protracted oxidative stress. Prostate.

[19] Chatterjee A., Gupta S. (2018). The multifaceted role of glutathione S-transferases in cancer. Cancer Lett..

[20] Kanwal R., Pandey M., Bhaskaran N., Maclennan G.T., Fu P., Ponsky L.E., Gupta S. (2014). Protection against oxidative DNA damage and stress in human prostate by glutathione S-transferase P1. Mol. Carcinog..

[21] Wu J.D., Lin D.W., Page S.T., Lundgren A.D., True L.D., Plymate S.R. (2009). Oxidative DNA damage in the prostate may predispose men to a higher risk of prostate cancer. Transl. Oncol..

[22] Miyake H., Hara I., Kamidono S., Eto H. (2004). Oxidative DNA damage in patients with prostate cancer and its response to treatment. J. Urol..

[23] Malins D.C., Johnson P.M., Barker E.A., Polissar N.L., Wheeler T.M., Anderson K.M. (2003). Cancer-related changes in prostate DNA as men age and early identification of metastasis in primary prostate tumors. Proc. Natl. Acad. Sci. USA.

[24] Glutathione, Reduced (GSH). Monograph. Alternative Medicine Review: A Journal of Clinical Therapeutic. https://www.ncbi.nlm.nih.gov/pubmed/11804544.

[25] Tsikas D. (2017). Assessment of lipid peroxidation by measuring malondialdehyde (MDA) and relatives in biological samples: Analytical and biological challenges. Anal. Biochem..

[26] Requena J.R., Fu M.X., Ahmed M.U., Jenkins A.J., Lyons T.J., Thorpe S.R. (1996). Lipoxidation products as biomarkers of oxidative damage to proteins during lipid peroxidation reactions. Nephrol. Dial. Transplant..

[27] Singhal S.S., Singh S.P., Singhal P., Horne D., Singhal J., Awasthi S. (2015). Antioxidant role of glutathione S-transferases: 4-Hydroxynonenal, a key molecule in stress-mediated signaling. Toxicol. Appl. Pharm..

[28] Srivastava D.S., Mittal R.D. (2005). Free radical injury and antioxidant status in patients with benign prostate hyperplasia and prostate cancer. Indian J. Clin. Biochem. Ijcb.

[29] Kosova F., Temeltas G., Ari Z., Lekili M. (2014). Possible relations between oxidative damage and apoptosis in benign prostate hyperplasia and prostate cancer patients. Tumour Biol..

[30] Mockett R.J., Bayne A.C., Kwong L.K., Orr W.C., Sohal R.S. (2003). Ectopic expression of catalase in Drosophila mitochondria increases stress resistance but not longevity. Free Radic. Biol. Med..

[31] Blum J., Fridovich I. (1985). Inactivation of glutathione peroxidase by superoxide radical. Arch. Biochem. Biophys..

[32] Jung K., Seidel B., Rudolph B., Lein M., Cronauer M., Henke W., Hampel G., Schnorr D., Loening S. (1997). Antioxidant Enzymes In Malignant Prostate Cell Lines and In Primary Cultured Prostatic Cells. Free Radic. Biol. Med..

[33] Freitas M., Baldeiras I., Proença T., Alves V., Mota-Pinto A., Sarmento-Ribeiro A. (2012). Oxidative stress adaptation in aggressive prostate cancer may be counteracted by the reduction of glutathione reductase. FEBS Open Bio.

[34] Kumar B., Koul S., Khandrika L., Meacham R.B., Koul H.K. (2008). Oxidative stress is inherent in prostate cancer cells and is required for aggressive phenotype. Cancer Res..

[35] Sfanos K.S., Yegnasubramanian S., Nelson W.G., De Marzo A.M. (2018). The inflammatory microenvironment and microbiome in prostate cancer development. Nat. Rev. Urol..

[36] Nelson W.G., De Marzo A.M., DeWeese T.L. (2001). The molecular pathogenesis of prostate cancer: Implications for prostate cancer prevention. Urology.

[37] De Marzo A.M., Marchi V.L., Epstein J.I., Nelson W.G. (1999). Proliferative inflammatory atrophy of the prostate: Implications for prostatic carcinogenesis. Am. J. Pathol..

[38] Henderson C.J., McLaren A.W., Moffat G.J., Bacon E.J., Wolf C.R. (1998). Pi-class glutathione S-transferase: Regulation and function. Chem. -Biol. Interact..

