# Supplementary material for: Oxidative Stress and Antioxidant Status in High-Risk Prostate Cancer Subjects
Source: Diagnostics (Basel). 2020 Feb 27;10(3):126. doi: 10.3390/diagnostics10030126 (PMC7151307; doi:10.3390/diagnostics10030126)
Supplement: Supplementary file 1 [file diagnostics-10-00126-s001.pdf]

Supplemental Table 1. Serum PSA values of healthy control and high risk subjects.

HEALTHY SUBJECTS		PSA VALUE
**Number**	**ID**	**ng/ml**
1	C-001	0.84
2	C-002	1.43
3	C-003	6.34
4	C-004	1.19
5	C-005	3.1
6	C-006	2.22
7	C-007	2.3
8	C-008	1.19
9	C-009	2.22
10	C-010	1.19
11	C-011	5.71
12	C-012	1.35
13	C-013	2.14
14	C-014	2.93
15	C-015	1.4
16	C-016	2.69
17	C-017	3.97
18	C-018	4.68
19	C-019	2.3
20	C-020	2.77

Range 0.84 to 6.34 ng/ml; Average 2.598 ± 1.53 ng/ml

HIGH-RISK SUBJECTS		PSA VALUE
**Number**	**ID**	**ng/ml**
1	HR-001	9.76
2	HR-002	21.27
3	HR-003	20.71
4	HR-004	17.77
5	HR-005	13.09
6	HR-006	34.44
7	HR-007	15.63
8	HR-008	27.46
9	HR-009	14.76
10	HR-010	13.73
11	HR-011	8.65
12	HR-012	15.0
13	HR-013	17.54
14	HR-014	26.34
15	HR-015	9.84
16	HR-016	7.32
17	HR-017	13.33
18	HR-018	15.23
19	HR-019	28.89
20	HR-020	15.55

Range 7.32 to 34.44 ng/ml; Average 17.315 ± 7.03 ng/ml
Control *versus* High-risk *P*-value < 0.0001
